# Timing of Prenatal Stress Exposure Predicts Infant Sympathetic Nervous System and Affective Responses

**DOI:** 10.1002/dev.70140

**Published:** 2026-03-08

**Authors:** Cecilia Martinez‐Torteya, Amy K. Nuttall, G. Anne Bogat, Joseph S. Lonstein, Maria Muzik, Kevin J. Grimm, Douglas A. Granger, Alytia A. Levendosky

**Affiliations:** ^1^ Department of Psychiatry University of Michigan – Michigan Medicine Ann Arbor Michigan USA; ^2^ Department of Human Development and Family Studies Michigan State University East Lansing Michigan USA; ^3^ Department of Psychology Michigan State University East Lansing Michigan USA; ^4^ Departments of Psychiatry, Obstetrics & Gynecology University of Michigan – Michigan Medicine Ann Arbor Michigan USA; ^5^ Department of Psychology Arizona State University Tempe Arizona USA; ^6^ Institute for Interdisciplinary Salivary Bioscience Research University of California – Irvine Irvine California USA; ^7^ Department of Pediatrics Johns Hopkins School of Medicine Baltimore Maryland USA

**Keywords:** prenatal stress, timing, SNS, sAA, fear, anger, infancy

## Abstract

Prenatal stress has broad detrimental consequences for neurodevelopment, with potential sensitive periods within gestation affecting specific developmental systems. We examined the effects of prenatal stress timing, level, and fluctuations on three markers of sympathetic nervous system activity: infant salivary alpha amylase (sAA), fear, and anger responses. In addition, we explored whether the effects of prenatal stress differed for boys and girls. We assessed 195 mother‐infant dyads (45% girls) from an ethnically diverse and economically disadvantaged community sample. Women reported perceived stress weekly from gestational week 14 to delivery. Dyads completed 6‐month postpartum in‐person assessments in which infants’ behavioral responses to two stressful tasks were coded and saliva collected. Machine learning analyses revealed that sAA and fear responses were predicted by increases in stress during the early third trimester (31–32 weeks) while increases in stress levels during mid‐ (21 weeks) and late‐gestation (38 weeks) predicted lower anger in response to a frustration task. Sex‐specific analyses pointed to different sensitive periods for boys and girls. Our findings emphasize the importance of collecting granular data during pregnancy to identify the epochs during which stress exposure is most pernicious, as well as the usefulness of assessing multiple indicators of infant biobehavioral reactivity to better capture the full toll of prenatal stress exposure.

## 1 Introduction

There is now substantial evidence to support gestation as a sensitive period during which maternal stress exposure negatively affects infant development (see reviews by Collins et al. 2024; Rodríguez‐Soto et al. 2021). The developmental origins of disease model (Barker, 1998) proposes risk for disease states based on biological alterations induced by early‐life adversity, including in gestation. Much of this research has focused on offspring hypothalamic–pituitary–adrenal (HPA) axis functioning as a mechanism that mediates risk for deficits in later biopsychosocial development (see reviews by Koss and Gunnar 2018; Tung et al. 2024). However, the other major stress response system, the autonomic nervous system (ANS), is frequently neglected. Prenatal stress may alter the sympathetic nervous system (SNS), one of the two branches of the ANS and a key substrate for the “fight or flight” response, as a way to prepare the fetus for the postnatal environment. When the SNS responds to stress or danger, the body prepares for action with emotions shaping behavioral responses, such that fear elicits avoidance, and anger elicits confrontation (Kamans et al. 2011). Chronic activation or alterations in SNS activity and its associated behavioral phenotypes (i.e., heightened fear and anger responses; Buchanan et al. 2010; Kreibig 2010; Payne et al. 2014) create risk for emotional and behavioral difficulties during childhood and further across the lifespan (Venn et al. 2025).

When confronted by stressors, the SNS is rapidly activated to release norepinephrine/noradrenaline that directly stimulates peripheral organs to assist in the earliest responses to threats. These organs are further stimulated indirectly by almost simultaneous norepinephrine/noradrenaline and epinephrine/ adrenaline release from the adrenal medulla that further contributes to this “fight or flight” response, which includes changes in cardiac output, body temperature, blood glucose levels, and immune function (Scott‐Solomon et al. 2021). While the SNS activates response during perceived danger, the parasympathetic nervous system (PNS) restores the body to a state of calm; both work in opposite ways to regulate many functions and parts of the body, and together comprise the ANS (Bleker et al. 2020).

Prenatal stress may alter the developing SNS via numerous mechanisms, including higher glucocorticoid receptor stimulation, altering early embryonic sympathetic cell differentiation and peripheral organ innervation (see Doupe et al. 1985; Michelsohn and Anderson 1992; Torres and Tucker 1995) and later on by dysregulating fetal sympathetic functioning (Mulder et al. 1994; Subtil et al. 2003). Our understanding of prenatal stress effects on the SNS is limited, though, because only a handful of studies evaluate their associations and results are mixed, with reports that stress predicts altered SNS activity in infants and young children (Fan et al. 2016; Weiss et al. 2024), significant effects exclusively for a subgroup of children (Vedhara et al. 2012), or null findings (Laurent et al. 2019). When measures that integrate SNS and PNS functioning are used, such as respiratory sinus arrythmia (RSA), results are also mixed, with reports that prenatal stress is associated with greater reactivity and failure to recover following a stressor (Bush et al. 2017; Gray et al. 2017) as well as null associations (Gao et al. 2022); moreover, other studies suggest RSA is independent from prenatal stress but moderates it's effects of infant negative affect (Peltola et al. 2017). Notably, these studies have not accounted for the timing of stress exposures during precise windows of fetal neurodevelopment.

The rapid and nonlinear development of the various fetal neural systems involved in the SNS response likely occurs during sensitive periods of prenatal life, thus increasing vulnerability to the effects of stress during those discrete epochs (Cerritelli et al. 2021). A few studies have examined timing‐dependent stress effects on the SNS and its behavioral phenotype: one study found that stress at 18 weeks gestation (but not 32) was associated with increased infant SNS activation (Vedhara et al. 2012), and two studies found that prenatal stress during the second trimester (but not first or third trimester) predicted infant negative affect (Kling et al. 2023; Rouse and Goodman 2014). In contrast, one study reported that “fluctuations in prenatal stress” exposure were the best predictor of infant negative affect (McNeil et al. 2023), suggesting that severity, timing, and fluctuations in prenatal stress may all be significant for infant SNS and behavioral responses. Findings related to sensitive periods of prenatal stress exposure for other important developmental outcomes are also mixed (see Collins et al. 2024 for review), but a majority of studies examining timing on infant stress responses report alterations in HPA axis activity after prenatal exposures during mid to late gestation (Davis et al. 2011; Levendosky et al. 2025).

In addition, a growing body of research suggests that prenatal stress affects males and females differently. There are important sex differences in fetal developmental processes, such that male fetuses experience higher levels of circulating androgens at various points, which inhibit corticotropin‐releasing hormone and adrenocorticotropic hormone production (Sze and Brunton 2020), and mature more slowly than females (Ellman et al. 2008; Hepper 2012). These sex differences in fetal brain developmental processes may be one of the underlying reasons for the common finding of sex differences in infant outcomes and the impact of early stress exposures. For example, female infants had more negative emotionality, fear, and aggression than males after exposure to prenatal stress (Braithwaite et al. 2017; Nolvi et al. 2019; Savory et al. 2020). Similarly, our prior research on the same sample studied herein found that, for girls, stress during mid‐gestation predicted difficult temperament and cortisol reactivity during infancy, whereas for boys, the sensitive period was in late‐gestation (masked). In contrast, the only study that evaluated sex‐specific impacts of *prenatal stress* on infant SNS reactivity reported that maternal anxiety and depression impacted boys’ reactivity, but not girls’ (Vedhara et al. 2012). Mixed findings are common in this literature, and that may be due to the confounding factor of the timing of prenatal stress assessment, which varies across studies.

### 1.1 The Current Study

Our study is the first rigorous evaluation of sensitive periods when the sympathetic and behavioral response to threats is most vulnerable to prenatal stress. We assessed physiological and emotional markers of the infant response to stressful situations (salivary alpha amylase [sAA]–a reliable marker of SNS activity; Granger et al. 2007) and observed fear and anger among 6‐month‐old infants. We examined fearful and angry distress as unique emotions, rather than consolidating them into a negative affect variable as most research on the effects of prenatal stress does (e.g., Davis and Sandman 2010). Because the SNS has a differential effect on these two emotions (Kamans et al. 2011), we were able to determine whether the prenatal timing of stress affected each differently in our 6‐month‐olds. We evaluated both physiological and behavioral markers of SNS activity because each of these indices is associated with social contextual factors (e.g., poverty; Hill‐Soderlund et al. 2015), peripartum maternal distress (Liu et al. 2019), and offspring internalizing and externalizing behaviors during infancy, childhood, and adolescence (Brooker et al. 2014; Funke et al. 2017; Gartstein et al. 2010; Kostyrka‐Allchorne et al. 2020; Lisonbee et al. 2010; Martinez‐Torteya et al. 2017; Messerli‐Bürgy et al. 2018).

To pinpoint the specific epochs of increased sensitivity for alterations in SNS and behavioral reactivity, we conducted weekly assessments of maternal perceived stress from 15 weeks of pregnancy to the time of giving birth. Although negative prenatal stress impacts have been related to stressful and traumatic life event exposure (Weiss et al. 2024; Martinez‐Torteya et al. 2016), depression (Kling et al. 2023; Vedhara et al. 2012), anxiety (Fan et al. 2016), as well as perceived stress (Davis et al. 2011), we focused on women's subjective ratings of stress, as meta‐analytic studies show stronger effects when studies use subjective stress as the predictor (Manzari et al. 2019). Our longitudinal, repeated‐measures design allowed us to determine the effects of (1) mean levels of maternal perceived stress during pregnancy, (2) fluctuations in stress levels during pregnancy, and (3) the timing (15 weeks to birth) of prenatal exposure to stress. In addition, most studies in this literature focus on perceived prenatal stress among women with low sociodemographic risk (e.g., Blair et al. 2011; Rash et al. 2016), but our study recruited a racially/ethnically diverse and economically disadvantaged sample, with overrepresentation of interpersonal violence and other adversities. Finally, many studies are confounded by single‐reporter data (i.e., mother's report; e.g., Braithwaite et al. 2017). In contrast, our study used an objective, multimethod assessment of infant outcomes.

Given the paucity of studies addressing timing‐specific prenatal stress effects on early SNS and emotional responses to threats, we explored the following research questions:

1. Do stress experienced during specific gestational weeks, fluctuations in stress during pregnancy, and/or mean levels of prenatal stress predict infant sAA reactivity, fearful distress, and angry distress after a psychosocial laboratory challenge? While the extant literature allowed us to hypothesize that more stress would be associated with more sAA reactivity, fear, and anger responses, we used an exploratory approach for the gestational week and fluctuation questions.
2. Are the effects of mean stress levels, fluctuations in stress during pregnancy, and sensitive periods different for boys and girls? We conducted exploratory sex‐specific analyses to address this question, as there is minimal research to guide specific hypotheses.

## 2 Methods

### 2.1 Participants

Participants were 195 women and infants (45% girls) enrolled in a longitudinal, NICHD‐funded study that evaluates the timing‐dependent effects of pregnancy stress on maternal and child outcomes (see [masked] for research protocol). We recruited women in early pregnancy (*M* = 17.75 weeks, SD = 2.62) from three metropolitan areas in [masked]. Women were recruited through flyers, social media, prenatal clinics/hospitals, and an OB‐GYN research registry. Women called the study offices and were screened for eligibility. Inclusion criteria were: (1) English fluency, (2) ages 18–34 years, (3) in a heterosexual romantic relationship during the pregnancy, and (4) no endocrine disorders or cancer treatments that would affect hormones, or nightshift work that would affect circadian rhythms. In the larger research project, women were oversampled for exposure to intimate partner violence (IPV), the most common pregnancy traumatic stressor (Mendez‐Figueroa et al. 2013). For those women who did not experience IPV, they had to have 2 or more other pregnancy stressors (food insecurity, financial strain, neighborhood violence, nonviolent family conflict) as well as income below the Medicaid cut‐off to be enrolled. Thus, the sample represents a vulnerable population with significant exposure to stress during pregnancy. All procedures were approved by (masked) IRB, the study's primary institution.

A total of 396 women who participated in the larger study were enrolled between August 20, 2017, and June 11, 2023. The sample for the present study consisted of 195 dyads for which women completed the weekly stress assessments and had children's AA or emotional response data. Women were aged 18 to 35 years (*M* = 27.08, SD = 4.46), and 51% identified as White, 38% Black, 7% as Latinx, and 4% with a different race/ethnicity (see Table 1). Thirty percent of women were nulliparous, 57% reported a high‐school or lower educational attainment, 59% were currently employed, and 73% reported living with a partner. These 195 women were not different from the rest of the sample regarding women's reports of fetal gestational age at study enrollment, maternal age, number of previous births, race/ethnicity, or income. Only 50 dyads completed assessments after the beginning of the COVID‐19 pandemic; 12% of mothers and 2% of infants were diagnosed with COVID‐19 during study participation. Infant outcomes were not associated with COVID‐related variables.

**TABLE 1:** Sample characteristics.

Maternal age	*M* = 27.08, SD = 4.46
Maternal race	51% White, 38% Black, 7% multiracial, 4% mther
Maternal education	12% < high school (HS), 45% HS or GED, 6% post‐HS technical degree, 25% college degree, 11% graduate degree
Maternal occupation	59% employed
Maternal relationship status	94% currently in a relationship; 73% living with a partner
Nulliparous mother	30%
Baby sex	45% girls, 55% boys
Baby race	39% White, 36% Black, 20% multiracial, 5% other
Family monthly income	*M* = $2937.51, SD = 2557.86
# people in household	*M* = 3.65, SD = 1.68

### 2.2 Procedures

Participants gave informed consent to participate in the study and completed their first assessment in project offices. Participants were then sent a link to the online weekly stress question. At 6 months postpartum, women and children came into project offices and completed a series of activities, and mothers answered questionnaires. Interviews began at 12:00 pm to standardize the timing of saliva collection. A baseline saliva sample was taken from the infants before administration of two stressful tasks from the Laboratory Assessment of Temperament Battery (arm restraint and mask task; Goldsmith and Rothbart 1999); infant and maternal responses to the tasks were videotaped for later coding. At three timepoints after the task (5‐, 20‐, and 40 min; Blair et al. 2011), additional saliva was collected. Mothers were taught how to collect their infant's saliva with a swab and then place it in a cryogenic vial. When scheduling the assessment visit, mothers were instructed not to give their baby anything to eat or drink other than water for an hour before the visit. Saliva samples were stored between −20°C and –80°C and shipped to the UCI Institute for Interdisciplinary Salivary Bioscience Research for assaying. Women were provided $50 for their participation, and children received a gift worth about $5.

### 2.3 Measures

#### 2.3.1 Weekly Stress Ratings

Stress was assessed weekly, online, beginning after the first pregnancy visit. If the participant did not have a smartphone, one was provided for free. Women had one week to complete the survey. If surveys were not completed the first day, women were sent reminders throughout the week. Women were provided $1 for each completed weekly survey, and we incentivized completion by providing additional money for completing consecutive weekly surveys. Women responded to the question, “How stressed did you feel this past week?” using a scale that ranged from 0 (no stress) to 6 (high stress). A single item to assess subjective stress has established construct and content validity (e.g., Elo et al. 2003) and has been associated with a valid and reliable scale of stress‐related emotional exhaustion (Glise et al. 2010). The single‐item stress question reduced participant burden for the weekly assessment.

#### 2.3.2 Salivary Alpha Amylase

Infant SNS functioning at 6‐month‐olds was measured by assessing sAA in saliva taken with a cotton swab before and three times after a laboratory stressor (details below). sAA can be obtained in a noninvasive manner and shows a reliable response to laboratory stressors during infancy (Davis and Granger 2009). All samples were assayed in duplicate using a commercially available kinetic reaction assay kit (Salimetrics, LLC) with a detection range of 2–400 U/mL. For four infants, all scores were below the lower level of detection, and their samples were excluded. In addition, one value for the baseline sample, three values for Sample 2, and three values from Sample 3 were truncated to the lower level of detection to minimize the effect of outliers. Four infants did not have a baseline score due to insufficient saliva, and three infants did not have poststressor scores due to samples not being collected or present in insufficient amounts. The peak‐baseline sAA value was calculated for the remaining 186 infants, and values were normally distributed (range = −80.36 to 151.21 U/mL; *M* = 10.36, SD = 26.61). Thirty‐seven percent of infants reached the peak at 5 min poststressor, 32% at 20 min poststressor, and 31% at 40 min poststressor.

#### 2.3.3 The Prelocomotor Laboratory Temperament Assessment Battery (LabTAB)

Two different LabTAB (Goldsmith and Rothbart 1999) tasks were videorecorded; each was appropriate for 6‐month‐olds—one elicited fear, the other anger. Before administration, the tasks were explained to the mother. The infant was placed in a highchair. Mothers could either watch from behind a one‐way mirror or stand/sit behind the infant. All mothers were instructed to stay out of the infants’ view, not speak, or touch the infant, to avoid well‐documented maternal buffering of infant cortisol responses (Hostinar et al. 2014)

For the fear task, a “stage” with a curtain was placed on a table in front of the infant. The research assistant was crouched below the curtain out of view of the child and, one at a time, showed the infant 4 scary masks. Each mask was shown for 10 seconds, then the curtain was closed for 5 seconds, and the next mask was shown, etc. Three infant behaviors were coded in 5 s intervals, and then a mean of intervals was calculated: Intensity of facial fear (0 = none to 3 = strong facial fear), intensity of distress vocalizations (0 = none to 5 = full intensity), and intensity of escape behavior (0 = none to 3 = vigorous escape behavior). To account for scale differences, we rescaled so that all three variables would range from 0 to 5. The fear variable for this study was the average of these three variables (*M* = 0.23, SD = 0.41, range = 0–1.83), following published research (Filippa et al. 2024; Shapiro et al. 2018).

Interrater reliability was established by coding subsets of videos until a weighted *κ* ≥ 0.7 was achieved. After a subset of reliability videos was coded, the coding team met to discuss discrepancies before coding another reliability set. This process was completed until reliability was achieved. After reliability was established, we coded a subsample of 26% of the videos, and intercoder reliability was good for all codes: intensity of facial fear (weighted *κ* = 0.78), intensity of distress vocalizations (weighted *κ* = 0.86), and intensity of escape (weighted *κ* = 0.83).

The anger task involved placing a novel and interesting toy on the tray of the highchair. The research assistant demonstrated how the toy worked and then let the infant play with the toy for about 15 to 30 s, making sure the infant was engaged with the toy. The interviewer then stood behind the infant, gently restraining the infant's arms and thus keeping the infant from accessing the toy. This position was held for 2 min or if the infant cried hard for 20 s—whichever came first. Three infant behaviors were coded for each 5 s interval, and a mean of intervals was then calculated: Facial anger (0 = none to 3 = strong facial anger), struggle (0 = none to 4 = high intensity struggle), and vocal distress (0 = none to 5 = full intensity cry/scream). To account for scale differences, we rescaled so that all three variables would range from 0 to 5. The anger variable for this study was the average of these 3 means of intervals (*M* = 0.70, SD = 0.91, range = 0–4.73), following published research (Filippa et al. 2024; Shapiro et al. 2018).

Interrater reliability on this anger task was established by coding subsets of videos until weighted *κ* ≥ 0.7 was achieved. After establishing reliability, we coded a subsample of 27% videos, and intercoder reliability was adequate for all codes: intensity of struggle (weighted *κ* = 0.70), intensity of facial anger (weighted *κ* = 0.79), and intensity of distress vocalizations (weighted *κ* = 0.92).

### 2.4 Analytic Approach

As is common in longitudinal studies with vulnerable populations, there was missing data (Nuttall et al. 2015). We accounted for missingness and conducted machine learning variable selection using a three‐step approach. First, we used multiple imputation to prepare datasets with no missingness. We were not able to identify any predictors of missingness and, therefore, did not include any auxiliary variables in this step. Second, we performed the machine learning variable selection process in each of these imputed datasets to identify which of the stress variables predicted infant outcomes. Machine learning uses algorithms to find patterns in the data; it was ideally suited for our study because of the large number of predictor variables involved (i.e., mean, standard deviation, and 27 weekly scores of pregnancy stress). Finally, we summarized the machine learning results across the imputed datasets. We repeated the machine learning variable selection process within each sex subsample to test our second hypothesis.

#### 2.4.1 Data Preparation

Missingness on the weekly stress variables was handled with multiple imputation (Rubin 1987) using Mplus (Muthén & Muthén, 2019). The Likert scale of the weekly stress variables was recognized during imputation using an ordered logistic regression model. Multiple imputation yielded 21 datasets with complete data.

Then, for each dataset, three types of stress variables were calculated. First, we calculated women's average stress level (one variable representing each woman's mean across weeks of gestation). Second, we calculated fluctuation in stress levels across gestation (one variable representing each woman's standard deviation across weeks). Third, we calculated a prenatal stress timing variable for each week (27 variables) by calculating a weekly deviation stress score defined as the difference between each participant's weekly stress scores and their mean stress scores. We computed deviation scores for evaluating the influence of each week because we expected a woman's own mean level of reported stress was a better comparison for her other weeks than a between‐persons approach that would suggest that all women rate their stress levels the same. Women's mean stress across pregnancy was 3.01, SD = 1.20 (range = 0–5.63). Women's weekly stress standard deviation across pregnancy was 1.41, SD = 0.37 (range = 0–2.63). Prenatal stress data were merged with the sAA and child behavioral data. This was done separately for each outcome such that participants with missingness on outcomes were removed before their respective data analysis, resulting in different sample sizes for each of the three models. The sample size for each model was *n* = 186 for sAA, *n* = 170 for fearful distress, and *n* = 157 for angry distress.

#### 2.4.2 Variable Selection

There were 29 explanatory variables in the variable selection algorithm. Variable selection was performed with the least absolute shrinkage and selection operator (LASSO) regression (Tibshirani 1996). LASSO regression penalizes the regression estimates, causing the regression estimates to shrink toward zero. When regression estimates shrink all the way to zero, the explanatory variable is removed from the model. The regression estimates for LASSO regression are found by minimizing the following loss function:

$$\sum_{i=1}^{N}{\left(y_{i}-{\hat{y}}_{i}\right)}^{2}+\lambda \sum_{j=1}^{p}\left|\beta_{j}\right|$$

where the first term is the residual sum of squares (typical loss function in ordinary least squares regression) and the second term is the penalty function, where *λ*, the tuning parameter, is multiplied by the sum of the absolute values of the regression coefficients ($\beta_{j}$). As *λ* increases, the regression estimates shrink toward zero, with estimates eventually shrinking to zero, resulting in a regression model with fewer estimated parameters. Different values of the tuning parameter *λ* are specified, and an optimal value is determined through k‐fold cross‐validation of the mean squared error. We used a modified version of LASSO regression, the adaptive LASSO (Zou 2006), which accounts for the different scales of the explanatory variables through the use of adaptive weights. Adaptive LASSO regression was conducted on each imputed dataset using repeated 10‐fold cross‐validation (Geisser, 1975) to select the optimal tuning parameter. The glmnet package in R was used with its built‐in k‐fold cross‐validation function (cv.glmnet) to conduct the Adaptive LASSO. Variable selection was run for the whole sample and again separately by child sex.

#### 2.4.3 Summary of Parameter Estimates

Across the 21 imputed datasets, we tracked the number of times each regression estimate was retained in the model (estimated at a nonzero value). For regression estimates that were retained in ≥10 of the imputed datasets, we calculated the average estimates across the 21 imputed datasets; these unstandardized regression coefficients are reported in the text. Heatmaps present standardized regression coefficients calculated by multiplying unstandardized coefficients by the ratio of the standard deviation of the associated predictor and the standard deviation of the outcome.

## 3 Results

### 3.1 Descriptive Statistics

Infant sAA means remained stable from baseline to 5, 20, and 40 min after the stressor (T1 *M* = 44.14, SD = 31.52; T2 *M* = 43.47, SD = 37.66; T3 *M* = 44.53, SD = 35.44; and T4 *M* = 43.87, SD = 36.46, all U/mL). The average peak (highest value from poststress samples) was nonetheless 54.47% larger than the baseline, and 41% of infants displayed a poststressor increase in sAA of 10% or more, which is almost identical to the percentage of “responders” using this criterion found in a prior study of sAA levels in 6‐month‐old infants given the same anger and fear tasks (Hibel et al. 2018). Bivariate correlations between the outcomes of interest revealed that fear and anger responses were moderately correlated (*r* = 0.30), but neither behavioral index was significantly correlated with sAA reactivity.

### 3.2 sAA Results (*n* = 186)

**FIGURE 1:**
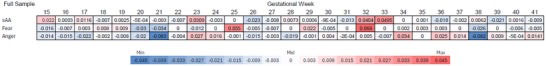
Heatmap of prenatal stress timing effects on sAA, fear, and anger reactivity. Note: Effects are color‐coded to represent negative values in blue and positive values in red, with darker shades representing stronger effects.

The Adaptive LASSO retained two explanatory variables in 10 or more of the 21 imputed datasets. The retained variables were the mother's weekly stress deviation scores on week 32 (retained in 12 imputations) and on week 33 (retained in 10 imputations). The estimated effects, averaged over the 21 imputations, were 0.79 (week 32) and 0.99 (week 33). Standardized effects are displayed in Figure 1.

### 3.3 Fearful Distress Results (*n* = 170)

The Adaptive LASSO retained two explanatory variables in 10 or more of the 21 imputed datasets. The retained variables were the mother's weekly stress deviation scores on week 25 (retained in 11 imputations; average *b* = 0.02) and week 32 (retained in 14 imputations; average *b* = 0.02).

### 3.4 Angry Distress Results (*n* = 157)

The Adaptive LASSO retained two explanatory variables in 10 or more of the 21 imputed datasets. The retained variables were the mother's weekly stress deviation scores on week 21 (retained in 14 imputations; average *b* = −0.05) and week 38 (retained in 13 imputations; average *b* = −0.05).

### 3.5 Within‐Sex Analyses

#### 3.5.1 Females (*n* = 86)

**FIGURE 2:**
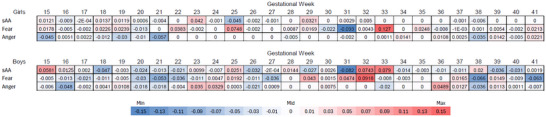
Heatmap of prenatal stress timing effects by sex. Note: Effects are color‐coded to represent negative values in blue and positive values in red, with darker shades representing stronger effects.

sAA: The Adaptive LASSO retained one explanatory variable in 10 or more of the 21 imputed datasets. The retained variable was the mother's weekly stress deviation score on week 25 (retained in 11 imputations). The estimated effect, averaged over the 21 imputations, was −0.74. Sex‐specific standardized effects are displayed in Figure 2.

Fearful distress: The Adaptive LASSO retained three explanatory variables in 10 or more of the 21 imputed datasets. The retained variables were the mother's weekly stress deviation scores on week 25 (retained in 10 imputations; average *b* = 0.02), week 31 (retained in 10 imputations; *b* = −0.02), and week 33 (retained in 10 imputations, *b* = 0.03).

Angry distress: The Adaptive LASSO retained zero explanatory variables in 10 or more of the 21 imputed datasets.

#### 3.5.2 Males (*n* = 105)

sAA: The Adaptive LASSO retained three explanatory variables in 10 or more of the 21 imputed datasets. The retained variables were the mother's weekly stress deviation scores on week 31 (retained in 11 imputations), week 32 (retained in 13 imputations), and week 33 (retained in 12 imputations). The estimated effects, averaged over the 21 imputations, were −1.68 (week 31), 1.47 (week 32), and 1.54 (week 33).

Fearful distress: The Adaptive LASSO retained two explanatory variables in 10 or more of the 21 imputed datasets. The retained variables were the mother's weekly stress deviation scores on week 32 (retained in 17 imputations; average *b* = 0.03), and week 38 (retained in 12 imputations; average *b* = −0.03).

Angry distress: The adaptive LASSO retained zero explanatory variables in 10 or more of the 21 imputed datasets.

## 4 Discussion

We examined the prospective influence of prenatal stress on 6‐month‐old infants' sAA, anger, and fear responses to laboratory tasks among a racially/ethnically diverse sample with high sociodemographic disadvantage. We used rigorous weekly assessment of maternal subjective stress levels to examine whether overall stress levels, stress timing, and/or fluctuations in stress throughout pregnancy affected infant outcomes. We also examined whether these relationships differed by infant sex.

Because we examined each woman's weekly stress score in the context of her overall stress levels, we were able to detect how weekly increases or decreases in pregnancy stress impacted infant biobehavioral regulation. Results showed that increases in stress during weeks 32 and 33 of pregnancy predicted more sAA reactivity, suggesting the earlier part of late gestation may be a particularly sensitive period for the effects of stress on infant “fight or flight” systems. Our findings coincide with the study by Posner et al.’s (2016), who found that maternal depressive symptoms during the earlier part of late gestation (week 34) were associated with increased fetal heart rate, a marker of SNS activity. Although many of the brain structures implicated in SNS function have accelerated growth early in gestation, the third trimester of pregnancy has also been proposed as a sensitive period due to the rapid maturation of the PNS, the counterpart to SNS activity (Cerritelli et al. 2021). Specifically, PNS signaling originating from the brainstem and transmitted through myelinated vagus nerve fibers shows maturational acceleration around 25 to 32 weeks of gestation, and our results may reflect disruption of the tail‐end of this process (Mulkey and du Plessis 2018). In addition, the significance of this time of rapid early third trimester for ANS development is also supported by evidence that preterm infants born at 32 weeks have stronger autonomic activity compared with preterm infants born even a week or two earlier (Hadas et al. 2021).

In our findings, the same precise gestational epoch (32 weeks) that was associated with increased sAA activity, along with mid‐gestation stress (week 25) were associated with increased infant expression of fearful distress in response to the laboratory threat task. Our findings are consistent with previous results that identify mid‐pregnancy stress as conveying vulnerability for negative affect (Kling et al. 2023; Rouse and Goodman 2014), which may be mediated via disrupted amygdalar development, given the rapid growth in amygdalar synaptogenesis during this period (Mulc et al. 2024). Our results also comport with a large longitudinal study documenting that maternal anxiety at 32 weeks is a stronger predictor of emotional problems in young children than maternal anxiety at 18 weeks of gestation (O'Connor et al. 2002). The effects of late gestation prenatal stress on infant fear responses reactivity may be mediated by altered infant amygdalar‐hypothalamic‐ and amygdalar‐prefrontal cortex functional connectivity (e.g., Scheinost et al. 2016; Humphreys et al. 2020), as well as intracortical functional connectivity (Thomason et al. 2021). Alternatively, the sensitive period at 32 weeks gestation may reflect heightened temporal lobe anatomical connections made with other cortical regions at this time (Thomason et al. 2015), or maturation of the anterior temporal lobe gyri (Prayer et al. 2006), which is involved in numerous types of emotional processing via its interconnections with the amygdala and frontal cortex (Wong and Gallate 2012).

Sensitive periods to stress exposure for anger were different than those for sAA and fear, highlighting the utility of assessing each emotion separately (as opposed to one negative emotionality variable). Notably, increases in perceived stress in mid‐ (week 21) and late‐gestation (week 38) were associated with *less* angry distress. Our findings thus conflict with Kling et al. (2023) report that observed infant negative affect after stress induction (i.e., still face paradigm) was associated with maternal depressive symptoms in the second (20 weeks) but not third trimesters (36 weeks). These seemingly discrepant results may be an artifact of methodological differences (e.g., measuring depressive symptoms vs. subjective stress levels; focus on general negative affectivity vs. infant anger). In addition, the differences in the gestational epochs relevant for when stress affected sAA reactivity and angry distress in our sample may reflect a decoupling of the systems underlying stress responsiveness and anger in infancy, which has been previously reported (Lewis and Ramsey 2005). The negative association between increases in prenatal stress during particular weeks and anger responses was unexpected; it is possible that, due to neuroplasticity, stress may sometimes be protective, accelerating development to prepare the infant to encounter a stressful environment (DiPietro et al. 2006). Alternatively, a functionalist perspective of emotions, in which anger serves the purpose of removing the barriers to the individual's goal (Witherington and Crichton 2007), would suggest that very low levels of anger in response to the frustrating task may also forecast future maladaptation.

Turning to the exploration of sex differences, our analyses offer preliminary support for distinct impacts of prenatal stress timing on sAA and fear responses for boys and girls. However, the findings are difficult to interpret because significant effects were present during middle and late gestation, both risk and protective epochs emerged, and no sex differences were evident for angry distress. For example, in girls, mid‐gestation (week 25) stress exposure is associated with less sAA reactivity, whereas for boys, the early third trimester emerged as a predictor of both lower (week 31) and higher (weeks 32 and 33) sAA reactivity. The pattern of findings mostly comports with our results of an earlier sensitive period for exposure to prenatal stress on infant HPA axis regulation in girls when compared with that of what was found for boys (masked). However, the sex‐specific association between prenatal stress and more sAA reactivity may suggest that boys are more susceptible to the effects of stress on their SNS during gestation than are girls. This conflicts with a review that found that girls, rather than boys, are more broadly vulnerable to prenatal stress‐related alterations in “neural structure, function, and neuroendocrine sensitivity” (Sutherland and Brunswasser 2018), but is consistent with the results of the only study to date that reports sex differences in SNS reactivity after prenatal stress exposure, in which only boys were affected (Vedhara et al. 2012).

Despite strengths including rigorous, frequent assessment of prenatal stress, multimethod assessment of infant outcomes, and recruitment of a diverse and economically disadvantaged sample, the limitations of our study should be considered. Firstly, our findings may not be generalizable to samples of women who experience low levels of stress; for example, some studies suggest that mild to moderate levels of stress may promote offspring development and later positive adaptation (DiPietro 2012), and those effects are more likely to be detected in less vulnerable groups. Second, we did not assess stress before 15 weeks of gestation, which was before we enrolled women into our study, thus preventing us from evaluating whether first‐trimester stress affected infant outcomes. In addition, we assessed relatively short‐term outcomes in the infants (i.e., 6 months postnatal). Additional research should examine whether prenatal stress leads to continued alterations in physiological and affective responses during childhood. Another limitation of our methods was that we did not include other factors that may contribute to alterations in sAA, fear, and anger responses as covariates (e.g., postpartum maternal stress or shared genetic vulnerability). However, while some of those associations are well documented, many previous studies have controlled for such factors and, like us, still find robust effects of prenatal stress (e.g., Bush et al. 2017; Rice et al. 2010), and our approach allowed us to identify specific periods of stress exposure as novel predictors. Lastly, we did not evaluate whether co‐activation of the SNS with other relevant stress‐response systems (e.g., HPA axis, PNS) was also altered by exposure to stress during specific epochs of gestation. Given that normative patterns of multisystem coordination (i.e., reciprocal, co‐activation) can be disrupted by prenatal stress exposure (Rash et al. 2016), this is a key direction for future research. Specifically, our findings suggest that the alterations in sAA levels may be related to disrupted maturation of the PNS; as such, additional research exploring prenatal stress effects on SNS/PNS coordination is needed to explore whether the sensitive periods we identified are also most disruptive for the multisystemic integrated stress response.

Nonetheless, our research is among the first to document differential sensitive periods for the impact of prenatal stress on infant SNS reactivity, fear, and anger responses, and the first one to utilize granular weekly measurement of subjective stress during gestation to clearly identify the epochs during which stress exposure is most pernicious. Our findings emphasize the importance of focusing on multiple indicators of infant biobehavioral regulation to better capture the full toll of exposure to prenatal adversity. Taken together, our results highlight the significance of the early third trimester of gestation (gestational weeks 31 to 33) as a sensitive period for stress‐related alterations in sAA and fear responses, and mid‐gestation (week 21) and very late gestation (week 38) contributing to decreased anger responses. Our findings also hint at sex‐specific periods of enhanced sensitivity for boys and girls, but additional research is needed to clarify whether these differences can be replicated.

Should our findings be replicated, there are important implications for health providers who treat and interact with pregnant women. Most pregnant women are likely to be involved in health care during the third trimester, even if they have not seen a health care provider previously. Our findings suggest this would be an ideal time to develop and implement preventive interventions to reduce maternal stress and have a salutary effect on early infant development.

## Funding

This study received support from an NIH grant 5R01HD085990 to Levendosky, Bogat, Lonstein, and Muzik.

## Ethics Statement

This research was conducted in adherence to ethical standards and approved by the appropriate Institutional Review Boards.

## Conflicts of Interest

Douglas A. Granger is the founder and chief scientific and strategy advisor at Salimetrics LLC and Salivabio LLC. The nature of these relationships is managed by the policies of the committees on conflict of interest at Johns Hopkins University School of Medicine and the University of California at Irvine. The remaining authors declare no conflicts of interest.

## Data Availability

The data that support the findings of this study are available from the corresponding author upon reasonable request.
